# Ecomorph or Endangered Coral? DNA and Microstructure Reveal Hawaiian Species Complexes: *Montipora dilatata/flabellata/turgescens* & *M. patula/verrilli*

**DOI:** 10.1371/journal.pone.0015021

**Published:** 2010-12-02

**Authors:** Zac H. Forsman, Gregory T. Concepcion, Roxanne D. Haverkort, Ross W. Shaw, James E. Maragos, Robert J. Toonen

**Affiliations:** 1 Hawai'i Institute of Marine Biology, University of Hawaii, Kaneohe, Hawaii, United States of America; 2 Cell Biology and Molecular Genetics, University of Maryland, College Park, Maryland, United States of America; 3 Biology Department, Grant MacEwan University, Edmonton, Canada; 4 Pacific/Remote Islands National Wildlife Refuge Complex, U.S. Fish and Wildlife Service, Honolulu, Hawaii, United States of America; Smithsonian Institution National Zoological Park, United States of America

## Abstract

*M. dilatata*, *M. flabellata*, and *M. patula* and 80 other scleractinian corals were petitioned to be listed under the US Endangered Species Act (ESA), which would have major conservation implications. One of the difficulties with this evaluation is that reproductive boundaries between morphologically defined coral species are often permeable, and morphology can be wildly variable. We examined genetic and morphological variation in Hawaiian *Montipora* with a suite of molecular markers (mitochondrial: COI, CR, Cyt-B, 16S, ATP6; nuclear: ATPsβ, ITS) and microscopic skeletal measurements. Mitochondrial markers and the ITS region revealed four distinct clades: I) *M. patula/M. verrilli,* II) *M.* cf. *incrassata*, III) *M. capitata*, IV) *M. dilatata/M. flabellata/M.* cf. *turgescens*. These clades are likely to occur outside of Hawai'i according to mitochondrial control region haplotypes from previous studies. The ATPsβ intron data showed a pattern often interpreted as resulting from hybridization and introgression; however, incomplete lineage sorting may be more likely since the multicopy nuclear ITS region was consistent with the mitochondrial data. Furthermore, principal components analysis (PCA) of skeletal microstructure was concordant with the mitochondrial clades, while nominal taxa overlapped. The size and shape of verrucae or papillae contributed most to identifying groups, while colony-level morphology was highly variable. It is not yet clear if these species complexes represent population-level variation or incipient speciation (CA<1MYA), two alternatives that have very different conservation implications. This study highlights the difficulty in understanding the scale of genetic and morphological variation that corresponds to species as opposed to population-level variation, information that is essential for conservation and for understanding coral biodiversity.

## Introduction

*Montipora dilatata* is thought to be one of the rarest corals known. It has only been found in Kāne'ohe Bay, O'ahu, and tentatively on Maro reef (*M.* c.f. *dilatata*) in the Northwestern Hawaiian Islands [1], [2]. In 2000, extensive surveys identified only three colonies of *M. dilatata* in Kāne'ohe Bay, where previously it was more abundant [1]. The decline of this coral has been attributed to high sensitivity to bleaching, in addition to other general threats (freshwater kills, sedimentation/habitat degradation, overgrowth by alien algae, and anchor/boat damage) that may impact a small population with a limited geographic distribution [1]. *M. dilatata* and Hawaiian congeners *M. flabellata*, *M. patula*, along with 80 other species of scleractinian coral have recently been petitioned to be listed for federal protection under the US Endangered Species Act [3]. *Montipora*, like many coral genera, has a high degree of morphological variation that can make confident identification problematic. There are some morphotypes of *M. dilatata* that are clearly distinct from other congeners; however, there are also intermediate morphotypes with some similarities to *M. capitata* or *M. flabellata*.

*Montipora* taxonomy (as with all reef building coral) is based on skeletal morphology, which recent studies have shown can be remarkably variable, with surprising examples of convergent evolution [4], [5], rapid evolution [6], and phenotypic plasticity [7], [8]. *Montipora* species are classified by arrangement and size of protrusions between corallites (e.g., “papillae” are smaller than corallites while “verrucae” are larger) and by colony form (laminar, encrusting, massive, and branching). There are often portions of colonies that are smooth and lack papillae or verrucae and colony form can be highly variable in some species, often within an individual colony [9], [10]. Because colony form, size, and arrangement of microskeletal characters are rarely discrete, taxonomists disagree about the number and names of *Montipora* species that occur in a geographic region such as Hawai'I [10]–[12]. Adding to taxonomic confusion, the conceptual nature of the coral species is a subject of debate, particularly regarding the permeability of reproductive boundaries between morphospecies, and the evolutionary significance and prevalence of interspecific hybridization (e.g. [9], [13]–[20]).

The confusing range of morphological variation observed in coral is widely thought to be associated with interspecific hybridization, with some clear and well-studied examples based on molecular and reproductive studies; most notably Atlantic *Acropora* [15], [18], (currently listed under the ESA) and the *Montastrea* complex [19], [20] (proposed for listing under the ESA). *Montipora* from Indonesia and the Great Barrier Reef were examined by van Oppen *et al*.[14] with the putative mitochondrial control region (hereafter referred to as CR), and the Pax-C intron. Although the mitochondrial genes resolved clear clades, several morphological species shared identical haplotypes and could not be separated, while some morphological species were clearly not monophyletic. The Pax-C intron data was generally consistent with the mitochondrial trees, with some exceptions which were interpreted to be evidence of past introgression from hybridization; however, contrasting rates of lineage sorting is an alternative explanation. Recent studies from other coral families provide examples of alternative interpretations of disagreement between morphology and genetics and discordance between genes. For example, Forsman *et al*.[6] found discordance between genes and morphology in *Porites* (some morphological species shared identical haplotypes, while others were not monophyletic); however, because of strong congruence between mitochondrial (COI, CR) and nuclear (ITS) markers, there was an alternative explanation: rapid evolution and possible intraspecific variation (phenotypic polymorphism) of gross colony-level skeletal morphology. Likewise, Flot *et al*. [21] examined two mitochondrial and four nuclear markers on five morphospecies of Hawaiian *Pocillopora*, and each gene showed varying levels of concordance with classification based on morphology: mitochondrial genes resolved four morphospecies, ITS-2 resolved two, while short, variable single copy nuclear markers (calmodulin, EF-1α, ATPs β) had highly divergent allelic copies that failed to resolve any groups. This pattern was more consistent with variable rates of lineage sorting among markers, than hybridization and introgression.

**Figure 1:**
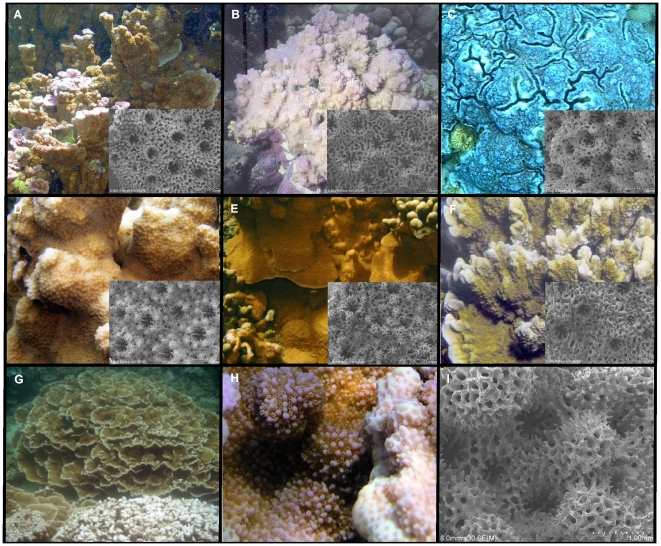
Hawaiian *Montipora* species and fine-scale morphology. (**A**) *M. dilatata*; (**B**) *M.* cf. *turgescens*; (**C**) *M. flabellata*; (**D**) *M. patula*; (**E**) *M. verrilli*; (**F**) *M.* cf. *incrassata*; (**G**) *M. capitata* (plating and branching morphs); (**H**) *M. capitata* (close up of branching morph); (**I**) *M.capitata* (SEM image).

There are many clear examples of hybridization in reef building corals, but it is not yet clear to what extent hybridization accounts for the observed patterns of molecular or morphological variation. Furthermore, it is conceptually and technically challenging to distinguish hybridization from intraspecific population-level variation, especially if there are more than three ‘species’ involved. The majority of studies on closely related coral taxa have had difficulty resolving closely related taxa, in part because mitochondrial markers evolve unusually slowly in Anthozoa [22]–[24], and in part because the scale of morphological and molecular genetic variation that corresponds to species as opposed to populations is not well understood. This study examines genetic and morphological variation in the genus *Montipora* in Hawai'i (Fig. 1), with the goal of determining if *M. dilatata* is genetically and morphologically distinct for corallite-level traits. We examined five mitochondrial genes, two nuclear genes, and a suite of morphological measurements to determine if there is congruence between molecular markers, and if there is concordance with measurements from SEM (Scanning Electron Microscope) images.

## Results and Discussion

**Figure 2:**
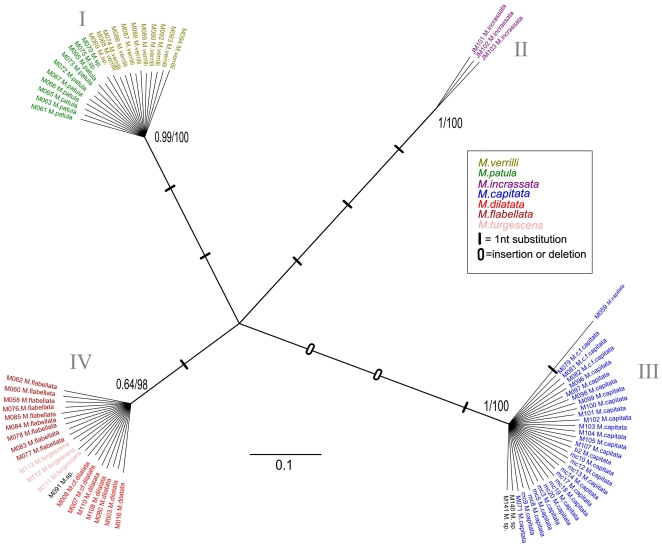
Bayesian inference tree of the mitochondrial control region for Hawaiian specimens. Confidence values are BI/ML, and species are color coded by morphospecies identification.

**Figure 3:**
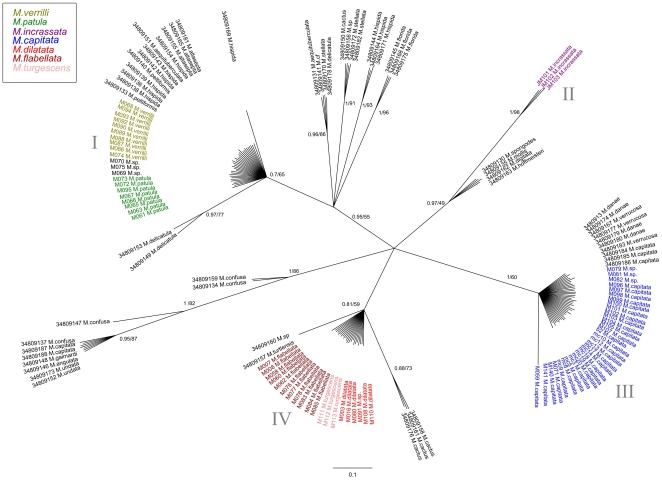
Bayesian inference tree of the mitochondrial control region for Hawaiian specimens and data from van Oppen et al.[**12**]. Confidence values are BI/ML, and species are color coded by morphospecies identification.

Two hundred and fifty-eight sequences were generated for this study (NCBI GenBank Accession #HQ246454-HQ246712). Comparisons with the GenBank database confirmed that the correct markers were amplified, with the highest sequence similarity to other *Montipora* sequences in the database. The majority of samples were sequenced for the mitochondrial control region (CR), (n = 74), and for ATPs β (n = 54) as they were the most highly polymorphic markers (Table S1). A subset of samples were chosen for further screening with additional mitochondrial genes; CO1 (n = 20) ATP-6 (n = 21),Cyt-B (n = 21),16S (n = 20) and the ITS region (n = 19), (Table S1). The Hawaiian CR sequences clustered into four strongly supported shallow clades, each separated from each other by three to seven fixed nucleotide differences (Fig. 2). There were no differences in haplotypes within the clades, with the exception of M059, which differed by a single base pair from other *M. capitata* samples. There were no differences between *M. dilatata*, *M. flabellata*, and *M.* cf. *turgescens* (Fig. 2, clade IV), or between *M. patula* and *M. verrilli* (Fig. 2, clade I). When compared to CR data from van Oppen et al. [14], *M. verrilli* and *M. patula* shared identical haplotypes with species outside of Hawai'i that also have papillae (*M. altasepta*, *M. hispida*, *M. peltiformis*, and *M. aequituberculata*) (Fig. 3, clade I). *M. capitata* shared identical haplotypes with species from outside of Hawai'i (*M. capitata*, *M. verrucosa*, and *M. danae*) that also have large verrucae (Fig.3, clade III). *M. dilatata*/*flabellata*/*turgescens* shared identical haplotypes with *M. turtlensis*, all species that have similar tubercular ridges (Fig. 3, clade IV). Although mitochondrial genes in corals may not evolve rapidly enough to resolve some species-level differences, it is interesting to note that these closely related genetic groups have similar fine-scale surface morphology. It is also interesting to note that colony level morphology and color can be extraordinarily variable, for example *M. capitata* contains colonies with thick branches, thin branches, plates, whirls, and hues of brown, red, orange and yellow. This clade also contained samples from deep water (∼60 m) that were consistent with descriptions of *Anacropora* or *M. tenuicaulis* Vaughan 1907 (long, thin branches with a smooth surface).

**Figure 4:**
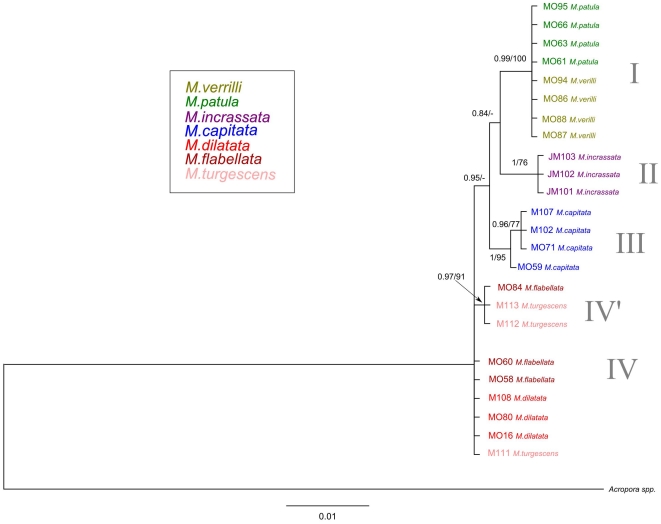
Bayesian inference tree of the concatenated mitochondrial dataset (ATP-6, COI, CYT-B, 16S) for Hawaiian specimens. Confidence values are BI/ML, and species are color coded by morphospecies identification.

Additional mitochondrial genes (ATP-6, COI, CYT-B, 16S) revealed no fixed differences corresponding to species within the clades (Fig. 4). The tree resolved an additional clade nested within the *M. dilatata*/*flabellata*/*turgescens* clade that differed only by a single nucleotide position in ATP-6, and was not fixed between species (Fig. 4, clade IV'). Unlike the CR tree, the concatenated mitochondrial data can be aligned and therefore rooted with an outgroup (*Acropora*) and can provide estimates for the divergence time between clades. Since Anthozoan mitochondrial genes evolve slowly, we have limited power to detect differences in recently diverged species. If we assume that mutations occur in a somewhat regular clock-like fashion, and that *Montipora* and *Acropora* diverged approximately 54 mya in accordance with the fossil record [25], then the rate of mitochondrial evolution is approximately 0.0005 bp/10^6^ years (which also corresponds to the rate estimated for Anthozoan mitochondrial genes by Helberg [24]). Since approximately 3,232 bp of mitochondrial DNA were surveyed, we may expect to find approximately 1.6 mutations for species that have been separated by one million years. Therefore, if *M. dilatata* is indeed a separate species, it has evolved recently.

**Figure 5:**
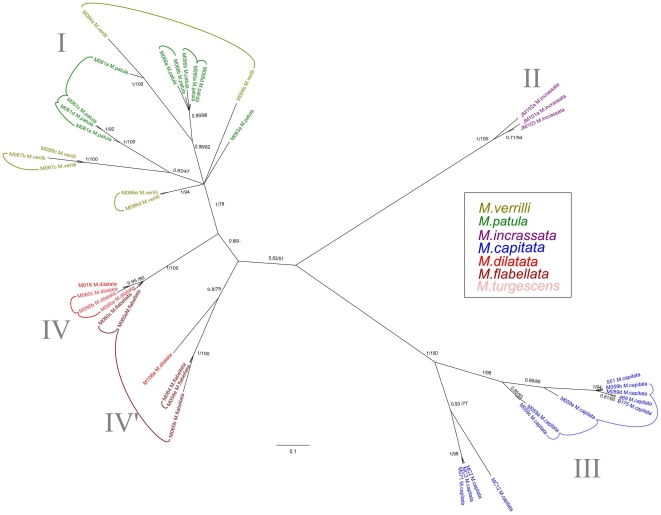
Cloned ITS region sequences for Hawaiian *Montipora*. Confidence values are BI/ML, and species are color coded by morphospecies identification. Colored lines indicate multiple sequences that were cloned from the same individual colony.

**Figure 6:**
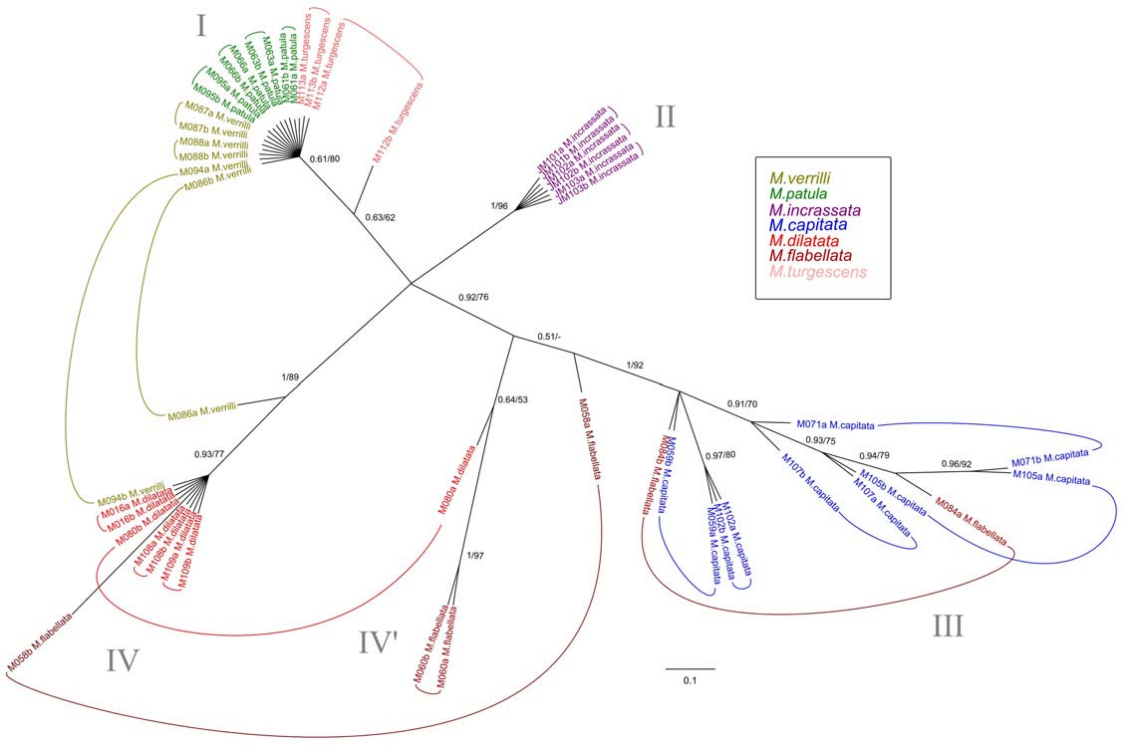
ATPs β haplotypes for Hawaiian *Montipora.* Confidence values are BI/ML, and species are color coded by morphospecies identification. Colored lines indicate haplotypes that were inferred from genotypic sequences using Phase [33].

The ITS tree (Fig. 5) resolved the same four groups as the CR tree, and an additional *M. dilatata*/*flabellata*/*turgescens* clade similar to the ATP-6/COI/CYT-B/16S tree in Fig. 4. The ITS region is a multi-copy marker with thousands of copies within a typical genome. Intragenomic variation (as indicated by colored lines in Fig. 5) was limited to occur within the mitochondrial clades. *M. dilatata* (M080a) and *M. flabellata* (M060e) shared an identical ITS sequence, indicating that these species have not been reproductively isolated over long evolutionary time scales. The ATPs β tree (Fig. 6) however, contrasted sharply with the mitochondrial and ITS trees. Although the same clades are somewhat discernable, there are some individuals that occur in unexpected clades, and some heterozygous individuals that bridge across several clades (Fig. 6). Since many of these same individuals were sampled across all gene regions and the ITS region tree is very similar to the mitochondrial trees it is unlikely that the discordance in the ATPs β is due solely to hybridization and introgression between lineages. Incomplete lineage sorting is a more likely explanation, especially when the following morphological results are taken into account.

**Figure 7:**
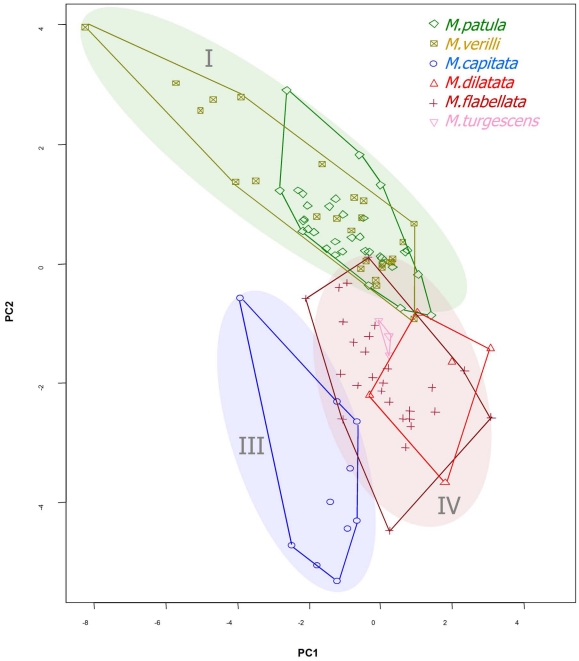
PCA plot of fine-scale morphological traits. A plot of the first two principal components color coded by species; background colors indicate genetic groups.

A PCA analysis was conducted on the 19 fine-scale measurements from 150 images of 47 specimens (Table S1, Table 1, Fig. 7). Thirty-one images were excluded from the analysis (including all *M.* cf. *incrassata* samples) because they did not contain measurable papillae, verrucae, or ridges (SEM resolution was too high to capture these features in all images). The morphological traits were highly variable (PC1 only accounted for 20.1 percent of the variation, and 12 principal components were necessary to encompass 90 percent of the variation). The size, shape, and density of papillae, verrucae, or ridges were primarily responsible for distinguishing the groups, as excluding these traits resulted in no clear groupings (data not shown). The morphological measurements strongly agreed with the genetic groups (MANOVA; Wilks test statistic; *p*<0.001), but all of the species within the genetic groups showed a high degree of overlap and could not be distinguished (MANOVA; Willks test statistic; *p* = 0.1912; Table 2). In other words, according to these measurements, *M. dilatata* could not be distinguished from *M*. *flabellata* or *M*. cf. *turgescens.* In addition, *M. patula* could not be distinguished from *M. verrilli*. The morphological measurements agree with the mitochondrial and ITS region data, which strengthens the case for incomplete lineage sorting as an explanation for discordance and incongruence with the ATPs β dataset. This interpretation is similar to Flot *et al*.'s [21] conclusion that mitochondrial genes corresponded well to morphological groups, followed by the ITS region, with little correspondence with single copy nuclear genes, which had considerable allelic diversity.

**Table 1:** List and definitions of morphological characters used.

**BC**	Distance from center of corallite to nearest verrucae, papillae or ridge
**NC**	Number of corallites
**NTP**	Number of verrucae, papillae or ridges
**MXDvSL**	Maximum corallite diameter (2N) divided by septal length (3N)
**LBp**	Proportion of maximum to minimum diameter of largest verrucae, papillae or ridge
**SBp**	Proportion of maximum to minimum diameter of smallest verrucae, papillae or ridge
**LPp**	Proportion of maximum to minimum diameter of largest pore
**SPp**	Proportion of maximum to minimum diameter of smallest pore
**MXD**	Maximum corallite diameter (average of 2 measurements)
**Dist**	Distance between corallites
**SL**	Maximum septa length (average of 3 measurements)
**LBmx**	Maximum diameter of largest verrucae, papillae or ridge
**LBmn**	Minimum diameter of largest verrucae, papillae or ridge
**SBmx**	Maximum diameter of smallest verrucae, papillae or ridge
**SBmn**	Minimum diameter of smallest verrucae, papillae or ridge
**LPmx**	Maximum diameter of largest pore
**LPmn**	Minimum diameter of largest pore
**SPmx**	Maximum diameter of smallest pore
**Spmn**	Minimum diameter of smallest pore

**Table 2:** Type II MANOVA of morphological measurements by group.

	Df	Test stat	Approx F	Df (groups)	Df (obs)	Pr(>F)
**Species**	1	0.92654	1.4498	7	128	0.1912
**Genetic group**	1	0.62219	11.1034	7	128	6.39E-11***

Tests: Wilks test statistic.

0.‘***’ 0.001 ‘**’ 0.01 ‘*’ 0.05.

Traditional fine-scale taxonomic characters that are fairly discrete generally agree with the genetic groups, for example; papillae (*M. patula* and *M. verrilli*); verrucae (*M. capitata*); variable short ridges (*M. dilatata*, *M. flabellata*, *M*. cf. *turgescens*) and nodular ridges (*M.* cf. *incrassata*) see [9]. These characters are important in distinguishing the genetic groups, however; these traits were not present to measure in all samples. For example, we collected a colony from a turbid and shaded environment (leeward mangroves) that was completely smooth, lacking any fine-scale surface features necessary for identification. CR sequences indicated the colony was in the *M. capitata* clade. We later observed fragments from this colony forming clear verrucae after five months in a holding tank. This observation indicates that verrucae can be phenotypically plastic (in this case present or absent depending on environmental conditions). It remains to be determined which environmental cue might initiate growth of verrucae; however, since these traits are the key for identifying species in this genus, the absence of these traits alone is not a reliable diagnostic feature. Colony-level morphology on the other hand is not evolutionarily conserved and varies wildly within the genetic groups. On the whole, these findings challenge the reliability of traditional taxonomy in this group, especially regarding gross colony-level skeletal morphology.

Until very recently, the study of evolutionary relationships among coral species relied solely on morphological characters; however, recent genetic evidence has called the validity of taxonomy by gross colony-level morphology into question [4]–[6]. In addition to genetics, studies on phenotypic plasticity in corals have revealed that fragments taken from the same colony can exhibit strikingly different growth forms in different environments [7], [8]. The extent of both genetic and morphological intraspecific variation in corals is poorly understood, as there are still relatively few evolutionary studies. This is clearly problematic, because morphological taxonomy is the current basis for estimating species distributions, abundance, and extinction risk. These factors complicate the understanding and management of potentially threatened coral species such as *M. dilatata.* This study identified no fixed genetic or fine-scale morphological differences between *M. flabellata*, *M.* cf. *turgescens*, and *M. dilatata*, or between *M. patula* and *M. verilli*. According to the CR data, the geographic ranges of these species complexes are likely to extend beyond Hawai'i into the central Pacific (e.g. *M. turgescens* has a broad distribution throughout the Indo Pacific [10], [26]. Species within these complexes are either actively interbreeding, or very closely related (e.g., within one million years). Hawai'i is one of the most isolated archipelagos, and evolutionary novelty in corals has been shown to be concentrated on the edge of species distributions [27]; perhaps *M. dilatata, M. patula,* and *M. flabellata* are in the early process of speciation. Now that these species complexes have been identified, work is needed to determine if the nominal species within each complex freely interbreed. Unfortunately, this study provides little guidance for determining if these specific species are valid and should be listed under the ESA, but perhaps more importantly; it highlights major gaps in the present understanding of species as opposed to population-level variation in corals. This study is an example of how knowledge of species boundaries in corals is not only necessary for understanding patterns and processes of biodiversity and evolution, but is essential for conservation.

## Materials and Methods

A set of 78 coral tissue samples (CA 2 cm^2^) were collected and examined for this study as approved of by the State of Hawaii Department of Land and Natural Resources Special Activity Permits 2009-101, PMNM-2007-033, and PMNM-2008-047. These samples represented 7 *Montipora* species from the Main and Northwestern Hawaiian Islands (Table S1). To ensure consistency, JEM confirmed the identification of the samples to species level (Fig. 1). DNA extractions followed a protocol developed previously in our laboratory [28]. Briefly, DNA was extracted from small pieces of coral tissue (5 mm^3^) by digestion for 2–3 h in 200 µL of DNAB (0.4 m NaCl, 50 mm Na_2_ EDTA pH 8.0) +1% SDS+10 µL proteinase K (10 µg/mL) on a shaker at 55°C. An equal volume of 2X CTAB (cetyltrimethyl ammonium bromide) +10 µL/mL β -mercaptoethanol was then added, and the tube was vortexed before being incubated at 65°C for an additional 20–25 min. An equal volume of chilled chloroform was added prior to vortexing and incubation on a rotating platform for 2–3 h at room temperature. Finally, the supernatant was precipitated with 95% EtOH, pelleted by centrifugation, and subsequently washed with 70% EtOH. DNA was resuspended in 50 µL deionized water before making 50 fold dilutions (approximate final concentration of ∼5 ng/µL) in dI water for subsequent use as template for Polymerase Chain Reaction (PCR).

In order to examine genealogical concordance, multiple loci were examined. PCR primers were based on conserved portions of aligned sequences from the National Center for Biological Information's (NCBI) GenBank database, and designed with the aid of Primer 3 v 0.4.0 [29], or based on previously published primers (Table 3). PCR was performed on a Bio-Rad MyCycler thermal cycler. Each 25 µL PCR contained 1 µL of DNA template, 2.5 µL of 10X ImmoBuffer, 0.1 µL IMMOLASE DNA polymerase (Bioline), 3 mm MgCl2, 10 mm total dNTPs, 13 pmol of each primer, and dI water to volume. Hot-start PCR amplification conditions varied slightly depending on the primer set used, but was generally: 95°C for 10 min (1 cycle), 95°C for 30 s, annealing temperature (2 degrees less than primer melting temperature, ranging between 50 and 60°C) for 30 s, and 72°C for 60 s (35 cycles) followed by a final extension at 72°C for 10 min (1 cycle). PCR products were visualized using 1.0% agarose gels (1X TAE) stained with Gelstar®. For the ITS region, PCR products were ligated into the PgemT-EZ® cloning vector (Promega Inc.) and transformed into JM109 competent cells following manufacturers recommendations. After blue/white colony selection, colonies were screened for the correct sized insert by PCR with the M13 vector primers and direct sequenced. PCR products for direct sequencing were treated with 2 U of exonuclease I and 2 U of shrimp alkaline phosphatase (Exo:SAP) using the following thermocycler profile: 37°C for 60 min, 80°C for 10 min. Treated PCR products were then cycle-sequenced using BigDye Terminators (PerkinElmer) run on an ABI-3130XL automated sequencer at the EPSCoR core genetics facility at HIMB. Resulting sequences were inspected and aligned using Geneious Pro 4.8.5 [30] to implement either ClustalW [31] or Muscle [32].

**Table 3:** List of primers used for this study.

Gene	Primer	Sequence (5′-3′)	Aprox. size	Reference	
mt16S	Z16Sf	CCTCGGCTGTTTACCAAAAA	790 bp	This study	
	Z16Sr	AACATCGAGGTCGCAAACAT		This study	
mtATP-6	ZATP6F	GGCTCTGATCGCCTTGACTA	534 bp	This study	
	ZATP6R	GGCCCACTTGCAACTAACAT		This study	
mtCyt-B	ZMcytbf	GGGACAGATGTTGTGCAATG	649 bp	This study	
	Zmcytbr	CCCCCAACAAAGGGATTAGT		This study	
mtCox-1	ZCOIF	TCAACTAATCATAAAGATATTGGTACG	609 bp	Forsman et al. 2009 [6]	
	ZCOIR	TAAACCTCTGGATGCCCAAA		Forsman et al. 2009 [6]	
mtCR	Ms FP2	TAGACAGGGCCAAGGAGAAG	650 bp	van Oppen et al. 2004 [12]	
	MON RP2	GATAGGGGCTTTTCATTTGTTTG		van Oppen et al. 2004 [12]	
nITS	ZITS1	TAAAAGTCGTAACAAGGTTTCCGTA	750 bp	Forsman et al. 2009 [6]	
	ZITS2	CCTCCGCTTATTGATATGCTTAAA T		Forsman et al. 2009 [6]	
nATPsβ	atpsbF2	CGTGAGGGAAATGATTTCTACCATGAGATGAT	280 bp	This study	
	atpsbR2	CGGGCACGGGCGCCGGGGGGTTCGTTCAT		Concepcion et al. 2009 [39]	
	atpsbF3	TGATTGTGTCTGGTGTAATCAGC		Concepcion et al. 2009 [39]	

mt  =  mitochondrial, n =  nuclear.

The majority of samples were examined with both the mt CR and ATPs β; however, 20 samples were examined in greater detail with a suite of additional markers (Table S1). Some ATPs β sequences were heterozygous at a few nucleotide positions as indicated by clear double peaks. Phase [33] as implemented in DNAsp 5.10.01 [34] was used to estimate the haplotypes for each heterozygous individual. Any samples for which mtDNA and nDNA disagreed were re-extracted, re-sequenced and/or cloned and sequenced. For all ITS and CR alignments, gaps were coded as present or absent using the “simple” gap coding method employed in GapCoder v1.0 [35]. The nucleotide substitution model was selected in Modeltest V.3.7 [36], selected by the Akaike information criterion. All phylogenetic analyses were performed with Bayesian Inference (BI) and Maximum Likelihood (ML). BI trees were generated with Mr.Bayes 3.1.2 [37], with 1,100,000 generations and a burnin of 110,000 generations, and ML trees were generated from RaxML[38]. Skeletal fragments (2–3 cm diameter) were cleaned, dried, mounted on stubs, and imaged with a Hitachi S-800 Field Emission Scanning Electron Microscope operated at 15 KV with a minimum resolution of 20 mm. Digital images were calibrated and measured in ImageJ V.1.40 (available at http://rsb.info.nih.gov/ij; developed by Wayne Rasband, National Institutes of Health, Bethesda, MD). Nineteen morphological characters were measured from 130 images of 47 specimens. Thirty-one images (including all *M. incrassata* images) were excluded from the final analysis because they did not include measurable papillae, verrucae, bumps or ridges within the 20 mm field of view. Principal components analysis (PCA) and discriminant function analysis were performed in R (2.10.1) with the candisc package for canonical discriminate analysis and MANOVA.

## Supporting Information

Table S1 Table of sample collection and sequencing information.
(DOC)
