# Supplementary material for: Ecomorph or Endangered Coral? DNA and Microstructure Reveal Hawaiian Species Complexes: Montipora dilatata/flabellata/turgescens & M. patula/verrilli
Source: PLoS One. 2010 Dec 2;5(12):e15021. doi: 10.1371/journal.pone.0015021 (PMC2996308; doi:10.1371/journal.pone.0015021)
Supplement: Table S1 — Table of sample collection and sequencing information. (DOC) [file pone.0015021.s001.doc]

**Table S1** Table of sample collection and sequencing information

| **Code** | **Species** | **Location** | **Collector** | **CR** | **ATPsβ** | **ITS** | **COI** | **ATP-6** | **Cyt-B** | **16S** | **morph** |
| --- | --- | --- | --- | --- | --- | --- | --- | --- | --- | --- | --- |
| JM101 | *M. c.f. incrassata* | Kawai, Hawai‘i | J.M. | ✓ | ✓ | ✓ | ✓ | ✓ | ✓ | ✓ | ✓ |
| JM102 | *M. c.f. incrassata* | Kawai, Hawai‘i | J.M. | ✓ | ✓ | ✓ | ✓ | ✓ | ✓ | ✓ | ✓ |
| JM103 | *M. c.f. incrassata* | Kawai, Hawai‘i | J.M. | ✓ | ✓ | ✓ | ✓ | ✓ | ✓ | ✓ | ✓ |
| M059 | *M. capitata* | Magic Isl. , Oahu | G.C.;R.H. | ✓ | ✓ | ✓ | ✓ | ✓ | ✓ | ✓ | ✓ |
| M071 | *M. capitata* | Lanikai, Oahu | G.C.;R.H. | ✓ | ✓ | ✓ | ✓ | ✓ | ✓ | ✓ | ✓ |
| M096 | *M. capitata* | Maro Reef, NWHI | I.B. | ✓ | ✓ |  |  |  |  |  |  |
| M097 | *M. capitata* | Mare Reef, NWHI | I.B. | ✓ | ✓ |  |  |  |  |  |  |
| M098 | *M. capitata* | Maro Reef, NWHI | I.B. | ✓ | ✓ |  |  |  |  |  |  |
| M099 | *M. capitata* | Pearl and Hermes, NWHI | G.C. | ✓ | ✓ |  |  |  |  |  |  |
| M100 | *M. capitata* | Kure Atoll, NWHI | G.C. | ✓ | ✓ |  |  |  |  |  |  |
| M101 | *M. capitata* | Lisianski, NWHI | G.C. | ✓ |  |  |  |  |  |  |  |
| M102 | *M. capitata* | Lisianski, NWHI | G.C. | ✓ | ✓ |  |  |  |  |  |  |
| M103 | *M. capitata* | Lisianski, NWHI | G.C. | ✓ | ✓ |  |  |  |  |  |  |
| M104 | *M. capitata* | Pearl and Hermes, NWHI | G.C. | ✓ | ✓ |  |  |  |  |  |  |
| M105 | *M. capitata* | Pearl and Hermes, NWHI | G.C. | ✓ | ✓ |  |  |  |  |  |  |
| M106 | *M. capitata* | Pearl and Hermes, NWHI | G.C. | ✓ |  |  |  |  |  |  |  |
| M107 | *M. capitata* | Kure Atoll, NWHI | G.C. | ✓ | ✓ |  |  |  |  |  |  |
| B3 | *M. capitata B.* | HIMB, Oahu | G.C. | ✓ |  |  |  |  |  |  |  |
| MC10 | *M. capitata B.O.* | HIMB, Oahu | G.A. | ✓ |  |  |  |  |  |  |  |
| MC17 | *M. capitata B.O.* | HIMB, Oahu | G.A. | ✓ |  |  |  |  |  |  |  |
| MC18 | *M. capitata B.O.* | HIMB, Oahu | G.A. | ✓ |  |  |  |  |  |  |  |
| MC19 | *M. capitata B.O.* | HIMB, Oahu | G.A. | ✓ |  |  |  |  |  |  |  |
| MC3 | *M. capitata B.O.* | HIMB, Oahu | G.A. | ✓ |  | ✓ |  |  |  |  |  |
| MC5 | *M. capitata B.O.* | HIMB, Oahu | G.A. | ✓ |  |  |  |  |  |  |  |
| MC9 | *M. capitata B.O.* | HIMB, Oahu | G.A. | ✓ |  |  |  |  |  |  |  |
| MC12 | *M. capitata B.R.* | HIMB, Oahu | G.A. | ✓ |  | ✓ |  |  |  |  |  |
| MC13 | *M. capitata B.R.* | HIMB, Oahu | G.A. | ✓ |  |  |  |  |  |  |  |
| MC14 | *M. capitata B.R.* | HIMB, Oahu | G.A. | ✓ |  |  |  |  |  |  |  |
| MC20 | *M. capitata B.R.* | HIMB, Oahu | G.A. | ✓ |  |  |  |  |  |  |  |
| MC8 | *M. capitata B.R.* | HIMB, Oahu | G.A. | ✓ |  |  |  |  |  |  |  |
| M081 | *M.* cf*. capitata* | Pukoo, Molokai | C.H.;Z.F. | ✓ |  |  |  |  |  |  | ✓ |
| M082 | *M.* cf*. capitata* | Pukoo, Molokai | C.H.;Z.F. | ✓ |  |  |  |  |  |  | ✓ |
| M111 | *M. cf. turgescens* | Pearl and Hermes, NWHI | M.T. | ✓ |  |  | ✓ | ✓ | ✓ | ✓ | ✓ |
| M112 | *M. cf. turgescens* | Lisiansky, NWHI | M.T. | ✓ | ✓ |  | ✓ | ✓ | ✓ | ✓ | ✓ |
| M113 | *M. cf. turgescens* | Pearl and Hermes, NWHI | M.T. | ✓ | ✓ |  | ✓ | ✓ | ✓ | ✓ | ✓ |
| M114 | *M. cf. turgescens* | Pearl and Hermes, NWHI | M.T. |  |  |  |  |  |  |  | ✓ |
| M016 | *M. dilatata* | Kaneohe bay, Oahu | C.H. | ✓ | ✓ | ✓ | ✓ | ✓ | ✓ | ✓ | ✓ |
| M080 | *M. dilatata* | Kaneohe bay, Oahu | C.H.;Z.F. | ✓ | ✓ | ✓ | ✓ | ✓ | ✓ | ✓ | ✓ |
| M108 | *M. dilatata* | Waikiki Aquarium | G.C. | ✓ | ✓ | ✓ | ✓ | ✓ | ✓ | ✓ |  |
| M109 | *M. dilatata* | Waikiki Aquarium | G.C. | ✓ | ✓ |  |  |  |  |  |  |
| M110 | *M. dilatata* | Waikiki Aquarium | G.C. | ✓ | ✓ |  |  |  |  |  |  |
| M058 | *M. flabellata* | Magic Isl. , Oahu | G.C.;R.H. | ✓ | ✓ | ✓ | ✓ | ✓ | ✓ | ✓ | ✓ |
| M060 | *M. flabellata* | Magic Isl. , Oahu | G.C.;R.H. | ✓ | ✓ | ✓ | ✓ | ✓ | ✓ | ✓ | ✓ |
| M062 | *M. flabellata* | Magic Isl. , Oahu | G.C.;R.H. | ✓ | ✓ |  |  |  |  |  | ✓ |
| M076 | *M. flabellata* | Kaneohe bay, Oahu | G.C.;R.H. | ✓ | ✓ |  |  |  |  |  | ✓ |
| M077 | *M. flabellata* | Kaneohe bay, Oahu | G.C.;R.H. | ✓ | ✓ |  |  |  |  |  | ✓ |
| M078 | *M. flabellata* | Kaneohe bay, Oahu | G.C.;R.H. | ✓ | ✓ |  |  |  |  |  | ✓ |
| M083 | *M. flabellata* | Kaneohe bay, Oahu | E.C. | ✓ | ✓ |  |  |  |  |  | ✓ |
| M084 | *M. flabellata* | Kaneohe bay, Oahu | E.C. | ✓ | ✓ | ✓ | ✓ | ✓ | ✓ | ✓ | ✓ |
| M085 | *M. flabellata* | Kaneohe bay, Oahu | E.C. | ✓ | ✓ |  |  |  |  |  | ✓ |
| M115 | *M. flabellata* | Midway, NWHI | M.T. |  |  |  |  |  |  |  | ✓ |
| M116 | *M. flabellata* | Midway, NWHI | M.T. |  |  |  |  |  |  |  | ✓ |
| M061 | *M. patula* | Magic Isl. , Oahu | G.C.;R.H. | ✓ | ✓ | ✓ | ✓ | ✓ | ✓ | ✓ | ✓ |
| M063 | *M. patula* | Magic Isl. , Oahu | G.C.;R.H. | ✓ | ✓ | ✓ | ✓ | ✓ | ✓ | ✓ | ✓ |
| M064 | *M. patula* | Lanikai, Oahu | G.C.;R.H. |  |  |  |  |  |  |  | ✓ |
| M065 | *M. patula* | Lanikai, Oahu | G.C.;R.H. | ✓ | ✓ |  |  |  |  |  | ✓ |
| M066 | *M. patula* | Lanikai, Oahu | G.C.;R.H. | ✓ | ✓ | ✓ | ✓ | ✓ | ✓ | ✓ | ✓ |
| M067 | *M. patula* | Lanikai, Oahu | G.C.;R.H. | ✓ | ✓ |  |  |  |  |  | ✓ |
| M072 | *M. patula* | Lanikai, Oahu | G.C.;R.H. | ✓ | ✓ |  |  |  |  |  | ✓ |
| M073 | *M. patula* | Lanikai, Oahu | G.C.;R.H. | ✓ | ✓ |  |  |  |  |  | ✓ |
| M095 | *M. patula* | Kaneohe bay, Oahu | C.H.;Z.F.;R.H. | ✓ | ✓ |  |  |  |  |  | ✓ |
| M069 | *M. sp.* | Lanikai, Oahu | G.C.;R.H. | ✓ | ✓ |  |  |  |  |  | ✓ |
| M070 | *M. sp.* | Lanikai, Oahu | G.C.;R.H. | ✓ | ✓ |  |  |  |  |  | ✓ |
| M075 | *M. sp.* | Lanikai, Oahu | G.C.;R.H. | ✓ | ✓ |  |  |  |  |  | ✓ |
| M079 | *M. sp.* | Kaneohe bay, Oahu | J.S. | ✓ | ✓ |  |  |  |  |  |  |
| M091 | *M. sp.* | Kaneohe bay, Oahu | C.H.;Z.F.;R.H. | ✓ | ✓ |  |  |  |  |  | ✓ |
| M068 | *M. verrilli* | Lanikai, Oahu | G.C.;R.H. | ✓ | ✓ |  |  |  |  |  | ✓ |
| M074 | *M. verrilli* | Lanikai, Oahu | G.C.;R.H. | ✓ | ✓ |  |  |  |  |  | ✓ |
| M086 | *M. verrilli* | Kaneohe bay, Oahu | C.H.;Z.F.;R.H. | ✓ | ✓ | ✓ | ✓ | ✓ | ✓ | ✓ | ✓ |
| M087 | *M. verrilli* | Kaneohe bay, Oahu | C.H.;Z.F.;R.H. | ✓ | ✓ | ✓ | ✓ | ✓ | ✓ | ✓ | ✓ |
| M088 | *M. verrilli* | Kaneohebay, Oahu | C.H.;Z.F.;R.H. | ✓ | ✓ | ✓ | ✓ | ✓ | ✓ | ✓ | ✓ |
| M089 | *M. verrilli* | Kaneohe bay, Oahu | C.H.;Z.F.;R.H. | ✓ | ✓ |  |  |  |  |  | ✓ |
| M090 | *M. verrilli* | Kaneohe bay, Oahu | C.H.;Z.F.;R.H. | ✓ | ✓ |  |  |  |  |  | ✓ |
| M092 | *M. verrilli* | Kaneohe bay, Oahu | C.H.;Z.F.;R.H. | ✓ | ✓ |  |  |  |  |  | ✓ |
| M093 | *M. verrilli* | Kaneohe bay, Oahu | C.H.;Z.F.;R.H. | ✓ | ✓ |  |  |  |  |  | ✓ |
| M094 | *M. verrilli* | Kaneohe bay, Oahu | C.H.;Z.F.;R.H. | ✓ | ✓ |  |  |  |  |  | ✓ |
| M140 | *M.* cf*. tenuicaulis* | ‘Au’au Channel | S.C | ✓ |  |  |  |  |  |  |  |
| M141 | *M.* cf*. tenuicaulis* | ‘Au’au Channel | S.C. | ✓ |  |  |  | ✓ | ✓ |  |  |

Check marks indicate successfully sequenced or morphologically measured samples. Abbreviations: G.C.= Greg Concepcion, R.H.= Roxanne Haverkort, Z.F.= Zac Forsman, E.C.= Evelyn Cox, C.H. = Cynthia Hunter, M.T.= Molly Timmers, I.B. = Iliana Baums, G.A.= Greta Abey, S.C.=Steve Coles. B = branching morphology, P = plating morphology, O = orange color morph, R = red color morph
